# Transcriptomic and metabolomic analyses reveal the molecular mechanisms by which long-day photoperiods promote flowering in *Gossypium hirsutum* L.

**DOI:** 10.3389/fpls.2025.1657595

**Published:** 2025-09-11

**Authors:** Ning Zhang, Yujie Liu, Yuli Lu, Zhonghua Zhou, Qiming Wang, Aiyu Liu, Xiaoju Tu

**Affiliations:** ^1^ College of Agronomy, Hunan Agricultural University, Changsha, China; ^2^ Yuelushan Laboratory, Changsha, China; ^3^ College of Bioscience and Biotechnology, Hunan Agricultural University, Changsha, China

**Keywords:** cotton (*Gossypium hirsutum L*.), photoperiod, flowering time, multi-omics, jasmonic acid (JA), flavonoid biosynthesis

## Abstract

Photoperiod is a crucial environmental cue that regulates flowering time in plants, playing a vital role in crop adaptability and early maturity. However, the molecular mechanisms underlying photoperiod-regulated flowering in cotton (*Gossypium hirsutum* L.) remain unclear. In this study, cotton plants were exposed to different photoperiod treatments during the seedling stage. Phenotypic evaluation, transcriptomic sequencing, and metabolomic profiling were integrated to systematically investigate the effects of photoperiod on flowering time and the associated molecular and metabolic regulatory pathways. The results showed that long-day treatments significantly accelerated budding and flowering in cotton, advancing by 20 and 17 days, respectively, compared to short-day conditions. Transcriptome analysis identified numerous differentially expressed genes (DEGs) involved in photoperiod response, hormone signaling, and metabolic regulation. Weighted Gene Co-expression Network Analysis (WGCNA) further revealed that key photoperiod-related genes, including *GhFKF1*, were upregulated under long-day conditions and formed co-expression networks with flowering regulators. Integrated transcriptomic and metabolomic analyses revealed significant enrichment in glycerophospholipid metabolism, α-linolenic acid metabolism, and flavonoid biosynthesis pathways. Long-day treatment suppressed the expression of key genes and precursors involved in jasmonic acid biosynthesis, while simultaneously upregulating genes involved in flavonoid biosynthesis, leading to increased accumulation of metabolites such as myricetin. Therefore, we propose a theoretical model in which long-day treatment during the seedling stage integrates hormonal and photoperiodic signals by upregulating the expression of the *GhFKF1* gene. This regulation may contribute to the initiation of flowering by simultaneously suppressing jasmonic acid biosynthesis and activating the flavonoid biosynthetic pathway. Our findings offer a theoretical foundation and a novel perspective for understanding the photoperiodic response and molecular mechanisms underlying early maturation in cotton.

## 1 Introduction

Cotton (*Gossypium hirsutum* L.) is a major global economic crop, playing a vital role in both agricultural and industrial systems. It provides natural fibers for the textile industry and generates by-products such as cottonseed oil and animal feed, which are widely used in textiles, pharmaceuticals, and the chemical industry. These products support the livelihoods of millions of people worldwide, especially in developing countries such as China, India, and Pakistan, where cotton is often referred to as “white gold” (Puspito et al., 2015). However, the full growth cycle of cotton—from planting to ginning—can extend up to 200 days, requiring substantial labor and input costs. This extended duration restricts crop rotation and intercropping with crops such as winter wheat and rapeseed, thereby limiting the efficient utilization of arable land resources (Luo et al., 2020; Ali et al., 2019). More importantly, the prolonged growth period increases cotton’s vulnerability to climate change, particularly during late developmental stages when extreme weather events can result in flower and boll abscission, reduced fiber quality, and significant yield losses (Hafeez et al., 2019). Therefore, reducing the growth period and developing early-maturing cotton varieties have become key strategies to improve their adaptability and cultivation stability.

Early maturity in cotton is governed by a complex polygenic regulatory network involving several phenotypic traits, including flowering time, the position of the first fruiting branch, and the total growth duration (Wu et al., 2024; Li et al., 2017). These traits are highly interrelated and collectively shape the developmental trajectory of cotton. Among these traits, flowering represents a critical developmental transition from vegetative to reproductive growth, regulated by both genetic factors and environmental cues. Photoperiod is one of the most vital ecological cues regulating flowering time in plants (Wang et al., 2024a). Studies have shown that plants perceive day length to adjust their internal circadian clock, thereby activating a cascade of signaling pathways that induce the expression of flowering-related genes and initiate reproductive organ development (González-Delgado et al., 2025). In the model plant *Arabidopsis thaliana*, the classical “GI–CO–FT” photoperiodic flowering pathway is well characterized. The circadian clock component GIGANTEA (*GI*) regulates the expression of CONSTANS (*CO*), which in turn activates the transcription of FLOWERING LOCUS T (*FT*), thereby triggering floral induction (Yang et al., 2024; Haiden et al., 2025). In long-day plants, *CO* expression peaks under photoperiods exceeding a critical threshold and cooperates with light signals to activate *FT*, promoting flowering (Yeang, 2013). Conversely, in short-day plants such as rice, the CO homolog HEADING-DATE1 (*Hd1*) is upregulated under short-day conditions, also leading to floral induction (Sun et al., 2022). The “GI–CO–FT” regulatory module has been validated in various crops, highlighting the evolutionary conservation of photoperiodic flowering networks (Haiden et al., 2025).

Cotton originated in tropical regions, and its wild species exhibit typical short-day plant characteristics, characterized by strong photoperiod sensitivity. However, modern cultivated varieties of *Gossypium hirsutum* L. have gradually adapted to diverse growing conditions through long-term artificial selection and are now considered photoperiod-insensitive, or day-neutral plants (Zhao et al., 2023). Nevertheless, cotton has not entirely lost its capacity to respond to photoperiodic cues. Studies have shown that expression of the cotton *FT* homolog (*GhFT*) peaks after 4–8 hours of light exposure, whereas in Arabidopsis, *AtFT* requires a 16-hour long-day condition to reach maximal expression (Guo et al., 2015; Freytes et al., 2021). These observations suggest that cotton retains a degree of photoperiodic regulatory capacity. A recent study demonstrated that long-day conditions significantly promote cotton flowering by modulating the expression of *GhFKF1*, a core component of the circadian clock, and its downstream flowering regulatory genes (Pan et al., 2024). These findings indicate that, even under modern cultivation practices, photoperiodic signals may still influence the reproductive transition of cotton at specific developmental stages. However, compared to model species such as Arabidopsis, rice, and soybean (Yang et al., 2024), our understanding of the molecular mechanisms underlying photoperiod-regulated flowering in cotton remains limited and lacks systematic insights. Although several studies have explored the roles of genes involved in photoperiod signal transduction and circadian rhythms in cotton flowering (Hao et al., 2021; Hua et al., 2025; Zhang et al., 2015), investigations into the regulation of flowering time in current cotton cultivars under different photoperiod treatments remain limited. Importantly, the existence of a photoperiod-sensitive “developmental window” during the seedling stage of cotton, which may regulate subsequent flowering through transcriptional and metabolic reprogramming, remains an open question.

Therefore, this study employs various photoperiod treatments during the seedling stage and integrates transcriptomic and metabolomic analyses to systematically investigate the regulatory effects of photoperiod on flowering time in cotton, as well as the underlying molecular mechanisms. This research provides important insights into the photoperiodic response of cotton, offering guidance for optimizing cultivation systems and breeding early-maturing varieties.

## 2 Materials and methods

### 2.1 Plant material and experimental design

The experimental material used was the conventional, early-maturing cotton variety XJ12-2, developed in China. Uniform, healthy seeds were sown in plastic pots (25.5 cm in diameter and 16.0 cm in height) filled with a 1:1 mixture of commercial seedling substrate and agricultural soil collected from a cotton-cultivated field in Liuyang, Hunan Province, China (28°18’N, 113°49’E). In conventional cotton cultivation, transplanting is typically performed at the three-leaf stage. Therefore, after emergence, the seedlings were transferred to a controlled-environment chamber and subjected to photoperiod treatments for 20 days. Watering was performed regularly to maintain soil moisture. Four photoperiod treatments were applied: (1) LL – continuous long-day treatment for 20 days (16 h light/8 h dark); (2) SS – continuous short-day treatment for 20 days (8 h light/16 h dark); (3) LS – long-day treatment for the first 10 days followed by short-day treatment for the last 10 days; and (4) SL – short-day treatment for the first 10 days followed by long-day treatment for the last 10 days. Each treatment consisted of 8 pots, totaling 32 pots. Twenty uniformly sized seeds were sown in each pot. Weak seedlings were removed at emergence to ensure uniform growth. Approximately 20 days after emergence, thinning was performed to retain two uniformly growing plants per pot, regardless of their flowering status. In the climate chamber, the light intensity was maintained at 350 μmol·m⁻²·s⁻¹, with a relative humidity of 70% and day and night temperatures of 30°C and 25°C, respectively. After completion of the photoperiod treatments, all cotton plants were transferred outdoors for acclimation to natural light. Following a 50-day acclimation period under field conditions (flowering stage), the fourth fully expanded leaf from the top of each plant was collected. Three uniformly growing plants per treatment were selected for sampling. Leaf samples were immediately frozen in liquid nitrogen and then stored at −80 °C for subsequent transcriptomic and metabolomic analyses.

### 2.2 Plant growth parameters

The budding and flowering times were recorded based on the criterion that 50% of the cotton plants in each pot had reached the respective stage. Three uniformly growing plants were selected from each pot, and their plant height and stem diameter were measured under natural light conditions after 0, 20, and 50 days of adaptation. Plant height was measured using a ruler with a precision scale of 1 mm, and stem diameter was measured using a digital caliper. Each measurement was performed in triplicate for all treatments.

### 2.3 Chlorophyll content and leaf color characterization

At 0, 20, and 50 days after transfer to natural light conditions, the relative chlorophyll content of the fourth fully expanded leaf from the top was determined using a SPAD-502 chlorophyll meter (Minolta Camera Co., Ltd., Japan). Simultaneously, a CR-10 PLUS colorimeter (Konica Minolta, Japan) was used to assess leaf color. Before measurements, the device was calibrated using a standard white reference plate. Leaf color parameters were recorded in the CIELAB color space, including L^*^ (lightness: 100 = white, 0 = black), a^*^ (positive = red, negative = green), and b^*^ (positive = yellow, negative = blue). Measurements avoided leaf veins and were taken at three different points on each leaf, with the average value used for analysis.

### 2.4 Transcriptomic analysis

Total RNA was extracted from 12 cotton leaves using TRIzol reagent (Invitrogen, Carlsbad, CA, USA), and genomic DNA contamination was removed using DNase I (TaKaRa, Japan). RNA integrity and purity were assessed using a 2100 Bioanalyzer (Agilent Technologies, CA, USA) and a NanoDrop ND-2000 spectrophotometer (Thermo Fisher Scientific, Madison, WI, USA). High-quality RNA was used to construct transcriptome libraries using the TruSeq™ RNA Sample Preparation Kit (Illumina, San Diego, CA, USA). RNA sequencing was performed on an Illumina NovaSeq 6000 platform at Wuhan Mytel Biotechnology Co., Ltd. (Wuhan, China). Raw reads were filtered and trimmed using *fastp* (https://github.com/OpenGene/fastp) to generate high-quality clean reads. Clean reads were aligned to the *Gossypium hirsutum* L. TM-1 reference genome (https://mascotton.njau.edu.cn/info/1054/1118.htm) using HISAT2. Transcript abundance was quantified using FPKM (Fragments Per Kilobase of transcript per Million mapped reads). Differentially expressed genes (DEGs) are filtered for |log2Fold Change| ≥ 1, and FDR<0.05, and KEGG enrichment analysis was performed on the DEGs to identify the key pathways.

### 2.5 Metabolomic analysis

Cotton leaf samples were freeze-dried using a vacuum lyophilizer (Scientz-100F), and then ground into a fine powder using a tissue grinder (30 Hz, 1.5 min). A total of 50 mg of the powdered sample was weighed and extracted with 1,200 μL of 70% methanol aqueous solution (pre-cooled to −20 °C) containing internal standards. The mixture was vortexed for 30 seconds every 30 minutes, for a total of six times. After centrifugation at 12,000 rpm for 3 minutes, the supernatant was collected and filtered through a 0.22 μm microporous membrane. The resulting filtrate was transferred to injection vials for ultra-performance liquid chromatography–tandem mass spectrometry (UPLC-MS/MS) analysis. UPLC-MS/MS analysis was performed using a Shimadzu LC-30A ultra-performance liquid chromatography system (Shimadzu, Kyoto, Japan) coupled with a TripleTOF 6600+ tandem mass spectrometer (SCIEX, Foster City, CA, USA).

All samples were analyzed using two LC-MS methods. One aliquot was analyzed under positive ion mode and separated using a Waters ACQUITY Premier HSS T3 column (1.8 µm, 2.1 mm × 100 mm). The mobile phase consisted of 0.1% formic acid in water (solvent A) and 0.1% formic acid in acetonitrile (solvent B), with the following gradient elution program: 5% B to 20% B over 2 minutes, ramped to 60% B over the next 3 minutes, then to 99% B within 1 minute and held for 1.5 minutes. The mobile phase was then returned to 5% B within 0.1 minute and equilibrated for 2.4 minutes. The analytical conditions were as follows: column temperature, 40 °C; flow rate, 0.4 mL/min; injection volume, 4 μL. Another aliquot was analyzed under negative ion mode, using the same chromatographic conditions and gradient elution program as described above.

Mass spectrometry was performed in information-dependent acquisition (IDA) mode, and data were acquired using Analyst TF 1.7.1 software. The instrument parameters were set as follows: ion source gas 1 (GAS1) and gas 2 (GAS2) at 50 psi; curtain gas (CUR) at 25 psi; interface temperature at 550 °C. The declustering potential (DP) was +60 V in positive mode and −60 V in negative mode. The ion spray voltage was set to +5000 V (ESI^+^) and −4000 V (ESI⁻). The TOF MS scan range was set from 50 to 1000 Da with an accumulation time of 200 ms, and dynamic background subtraction was enabled. For MS/MS scans, the range was 25–1000 Da with an accumulation time of 40 ms, collision energy ±30 V, and a collision energy spread of 15 V. Resolution was set to UNIT, with a maximum number of monitored ions of 18, a mass tolerance of 50 ppm, and a signal intensity threshold of 100 cps.

Raw mass spectrometry data were converted to mzXML format using ProteoWizard and subsequently processed with the XCMS software for peak detection, alignment, and retention time correction. Metabolic features missing in more than 50% of the samples were excluded. Missing values were imputed using the k-nearest neighbor (KNN) algorithm, and peak intensities were normalized using support vector regression (SVR). The remaining features were annotated by matching against public databases, including KEGG and HMDB, as well as using the metDNA approach. Metabolite identification was primarily based on MS/MS fragmentation spectra, complemented by precursor ion information and retention time comparisons with spectral libraries. Only metabolites with a total match score > 0.5 and a coefficient of variation (CV) < 0.5 in quality control (QC) samples were retained for downstream analysis.

QC samples were prepared by pooling equal aliquots from all individual extracts to assess instrument stability and data reproducibility. During the analysis, one QC sample was injected after every 10 experimental samples to ensure consistency and reliability. The identified metabolites were subsequently mapped to the KEGG pathway database (http://www.kegg.jp/kegg/pathway.html) for pathway annotation and enrichment analysis.

Unsupervised principal component analysis (PCA) and orthogonal partial least squares discriminant analysis (OPLS-DA) were performed to assess the metabolomic data. Differential metabolites were identified based on Variable Importance in Projection (VIP) scores from the OPLS-DA model, using the following thresholds: |log_2_Fold Change| ≥ 1, VIP > 1, and p-value < 0.05.

### 2.6 RT-qPCR validation

To validate the RNA sequencing (RNA-seq) results, we randomly selected 10 genes and analyzed their transcriptional levels by quantitative real-time PCR (qRT-PCR). Gene-specific primers were designed using Primer Premier 6.0 (**Supplementary Table S1**), and qRT-PCR amplification was performed on a Roche LightCycler 480 system (Roche, Switzerland) using the Talent qPCR Master Mix Kit (Tiangen Biotechnology, China). Vactin was used as the internal reference gene, and the relative expression levels of target genes were calculated using the 2^−ΔΔCt^ method.

### 2.7 Statistical analysis

Statistical analyses were conducted using SPSS software 23.0 (IBM Corp., Armonk, NY, USA). The significance was identified using one-way analysis of variance (ANOVA) with Tukey’s test (p < 0.05). All values are expressed as the mean ± standard deviation. The transcriptome and metabolome visualizations (Venn diagrams, heatmaps, PCA plots, etc.) were generated using an online platform (https://cloud.metware.cn). GraphPad Prism 10.0 (GraphPad Software, Boston, MA, USA) and Adobe Illustrator 2024 (Adobe Inc., San Jose, CA, USA) were utilized for other data visualization.

## 3 Results

### 3.1 Plant phenotype, chlorophyll content, and leaf color

**Figure 1:**
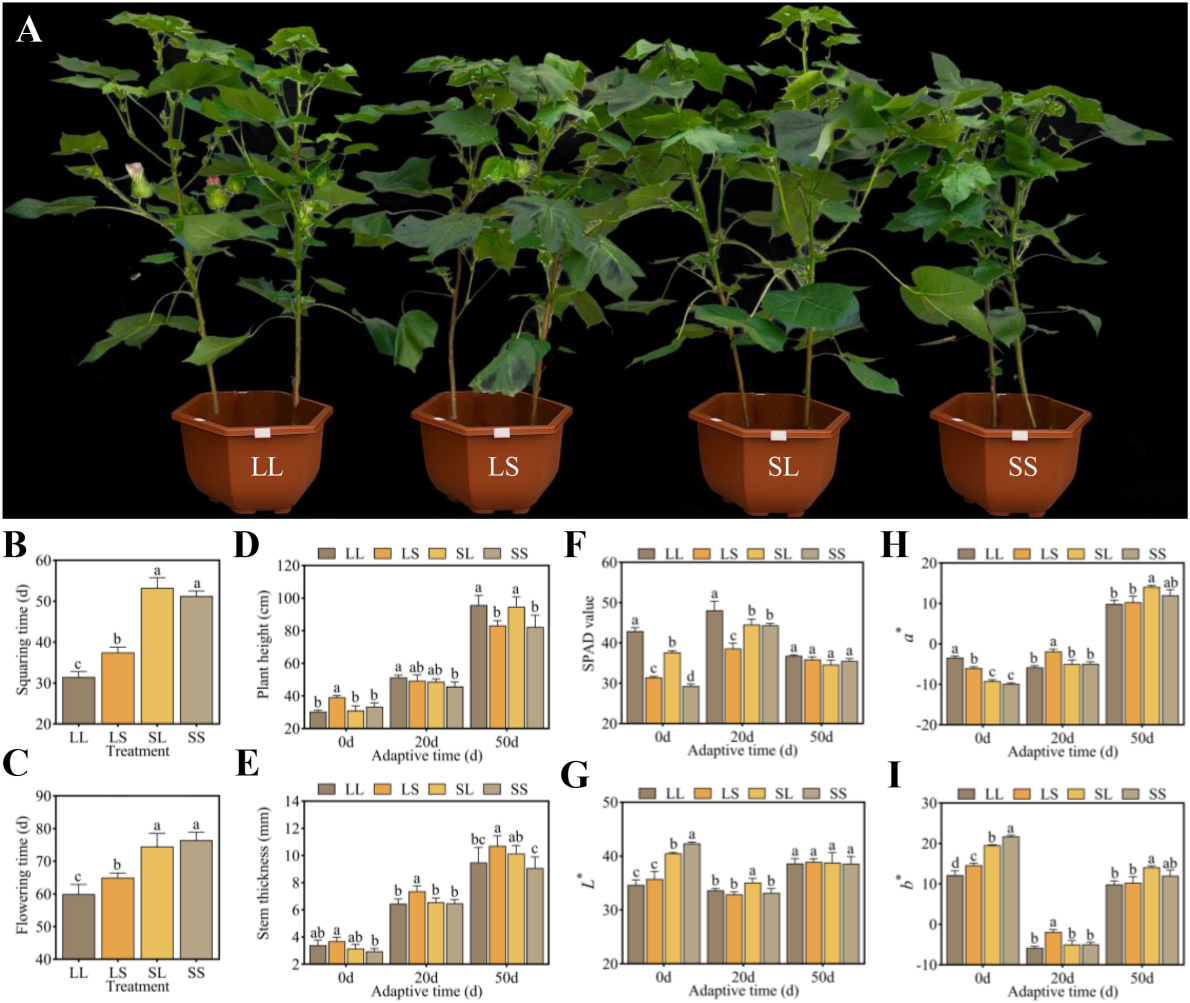
Effects of different photoperiod treatments at the seedling stage on cotton growth process, growth characteristics, and leaf physiological indexes. **(A)** Comparison of cotton phenotypes under different photoperiod treatments. **(B)** Squaring time. **(C)** Flowering time. **(D)** Plant height. **(E)** Stem thickness. **(F)** SPAD value. **(G)** *L* ^*^. **(H)** *a* ^*^. **(I)** *b* ^*^. Different lowercase letters indicate significant differences between treatments. The significance was identified using one-way analysis of variance (ANOVA) with Tukey’s test (p < 0.05).

To assess the effects of photoperiod treatments during the seedling stage on *Gossypium hirsutum* L., the growth dynamics and morphological parameters of cotton were recorded. The LL treatment led to the earliest bud emergence and flowering, significantly shortening the budding period by 6–20 days and the flowering period by 5–17 days compared with LS, SL, and SS treatments. Furthermore, LS treatment also significantly advanced both budding and flowering compared with SL and SS treatments (**Figures 1A–C**). After 50 days of natural light adaptation, plants under LL and SL treatments exhibited significantly greater height than those under LS and SS treatments (**Figure 1D**). Stem thickness remained highest in LS treatment throughout the adaptation period, whereas the SS treatment consistently showed the lowest values (**Figure 1E**). Relative chlorophyll content was estimated using SPAD values. During the early phase (0–20 days), LL treatment exhibited higher SPAD values, showing increases of 13.93%–46.57% and 7.93%–24.58% relative to LS, SL, and SS treatments, respectively. By day 50, SPAD values converged across all treatments (**Figure 1F**). In terms of leaf color, LL and LS treatments exhibited lower L^*^ (darker) and b^*^ (bluer) values, but higher a^*^ (redder) values at days 0 and 20. By day 50, no significant differences were observed in L^*^ values among treatments, whereas a^*^ and b^*^ values peaked in the SL treatment, significantly exceeding those in LL and LS treatments (**Figures 1G–I**). These results suggest that LL and LS treatments promoted developmental progression and accelerated flowering in cotton. Moreover, LL treatment maintained higher chlorophyll content throughout the adaptation period. Notably, SL and SS treatments showed favorable leaf color traits during the early adaptation phase, indicating better physiological status than LL and LS treatments.

### 3.2 Transcription analysis

**Figure 2:**
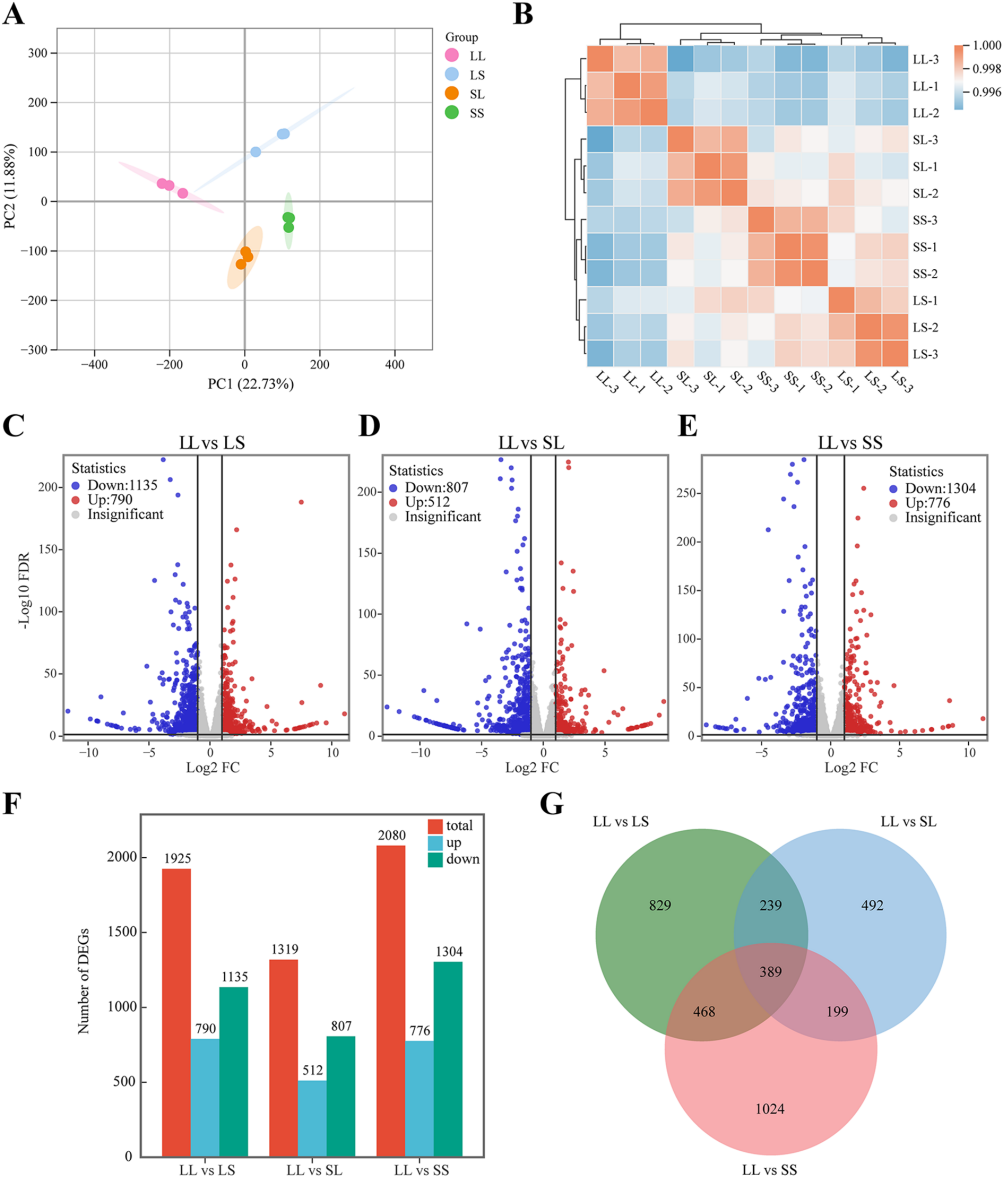
Transcriptome analysis of cotton leaves. **(A)** Principal component analysis of transcriptome data. **(B)** Correlation analysis of samples. **(C–E)** Volcano plot for different treatment groups. **(F)** Histogram of the number of DEGs. **(G)** Venn diagram of DEGs.

RNA sequencing was conducted on 12 leaf samples from four photoperiod treatment groups to elucidate the molecular mechanisms underlying the effects of seedling-stage photoperiod on cotton growth and development. After filtering out low-quality reads from the raw data, a total of 47,848,788 to 56,732,736 clean reads were obtained per sample. Of these, more than 97.76% were successfully mapped to the reference genome (*Gossypium hirsutum* L. acc. TM-1). All samples exhibited Q20 and Q30 values exceeding 98.23% and 94.51%, respectively, with GC contents ranging from 44.85% to 44.98% (**Supplementary Table S2**), indicating high sequencing quality. Principal component analysis (PCA) revealed distinct clustering of samples according to treatment groups (**Figure 2A**), while correlation analysis demonstrated high consistency among the three biological replicates (**Figure 2B**), confirming the reliability and reproducibility of the transcriptomic data. Differentially expressed genes (DEGs) were identified using the criteria |log_2_Fold Change| ≥ 1 and FDR < 0.05. Compared with the LS treatment, the LL treatment resulted in 790 upregulated and 1,135 downregulated genes (**Figure 2C**). Relative to the SL treatment, the LL treatment yielded 512 upregulated and 807 downregulated genes (**Figure 2D**). When compared with the SS treatment, the LL treatment led to 776 upregulated and 1,304 downregulated genes (**Figure 2E**). A Venn diagram analysis further revealed 389 DEGs commonly regulated across all three pairwise comparisons (**Figure 2G**). These results demonstrate that photoperiod treatments applied during the seedling stage exert significant regulatory effects on gene expression in *G. hirsutum*.

**Figure 3:**
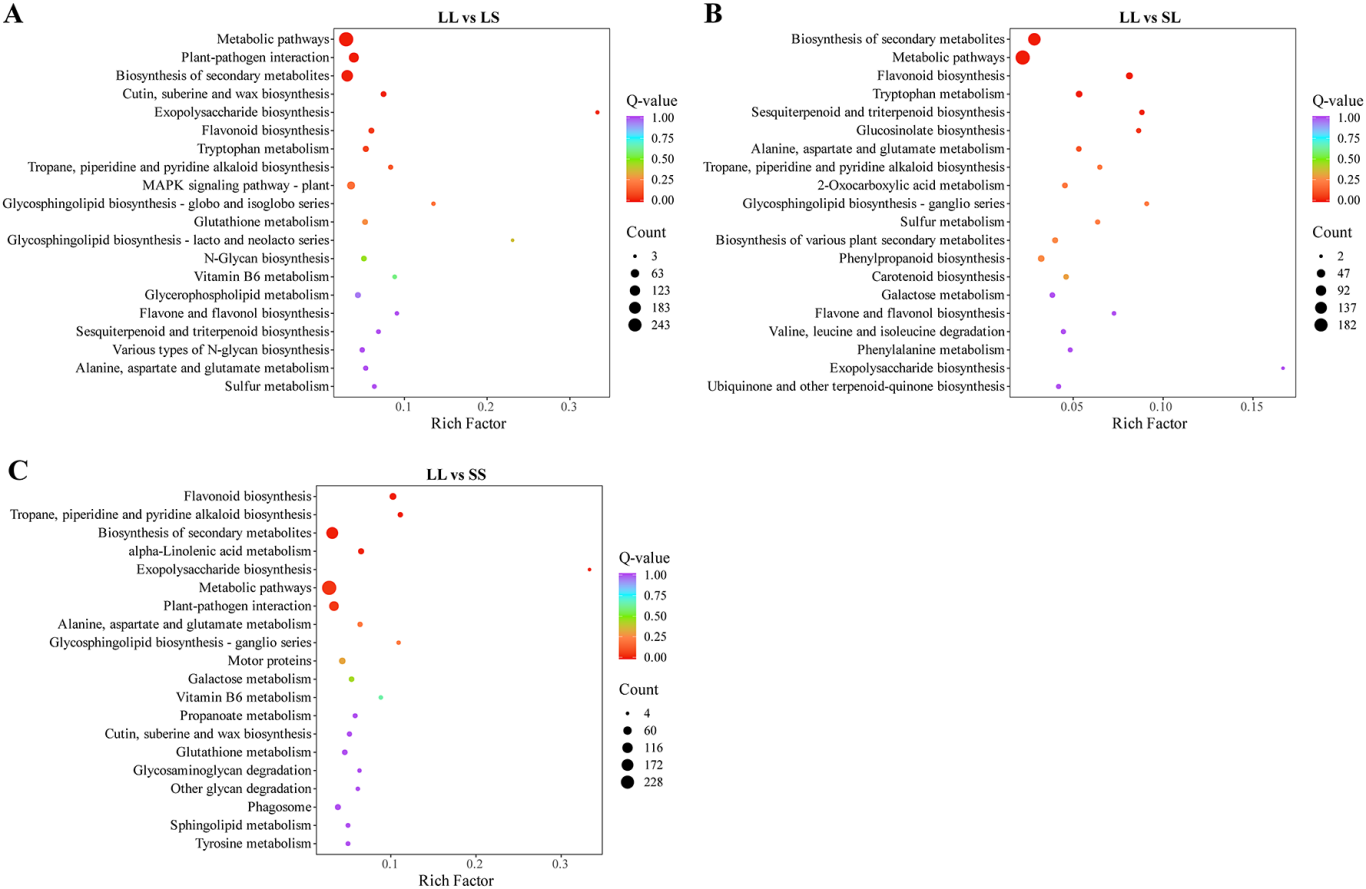
KEGG analysis of differential genes in three comparison groups. **(A)** KEGG analysis of differential genes in the LL vs LS group. **(B)** KEGG analysis of differential genes in the LL vs SL group. **(C)** KEGG analysis of differential genes in the LL vs SS group.

**Figure 3** presents the results of the KEGG enrichment analysis for differentially expressed genes (DEGs) across photoperiod treatment comparisons. In the LL vs. LS group, DEGs were predominantly enriched in pathways such as “Biosynthesis of secondary metabolites,” “Cutin, suberin and wax biosynthesis,” and the “MAPK signaling pathway – plant,” along with involvement in “amino acid metabolism” and “glycolipid biosynthesis” (**Figure 3A**). For the LL vs. SL group, significantly enriched pathways included “Flavonoid biosynthesis,” “Sulfur metabolism,” “Carotenoid biosynthesis,” as well as “amino acid degradation” and “vitamin metabolism” (**Figure 3B**). In the LL vs. SS group, DEGs were primarily enriched in “Flavonoid and alkaloid biosynthesis,” “α-Linolenic acid metabolism,” “Galactose metabolism,” and pathways related to “lipid and polysaccharide degradation” (**Figure 3C**). Notably, “Flavonoid biosynthesis” and “Tropane, piperidine and pyridine alkaloid biosynthesis” pathways were significantly enriched across all three comparisons.

### 3.3 Weighted gene co-expression network analysis

**Figure 4:**
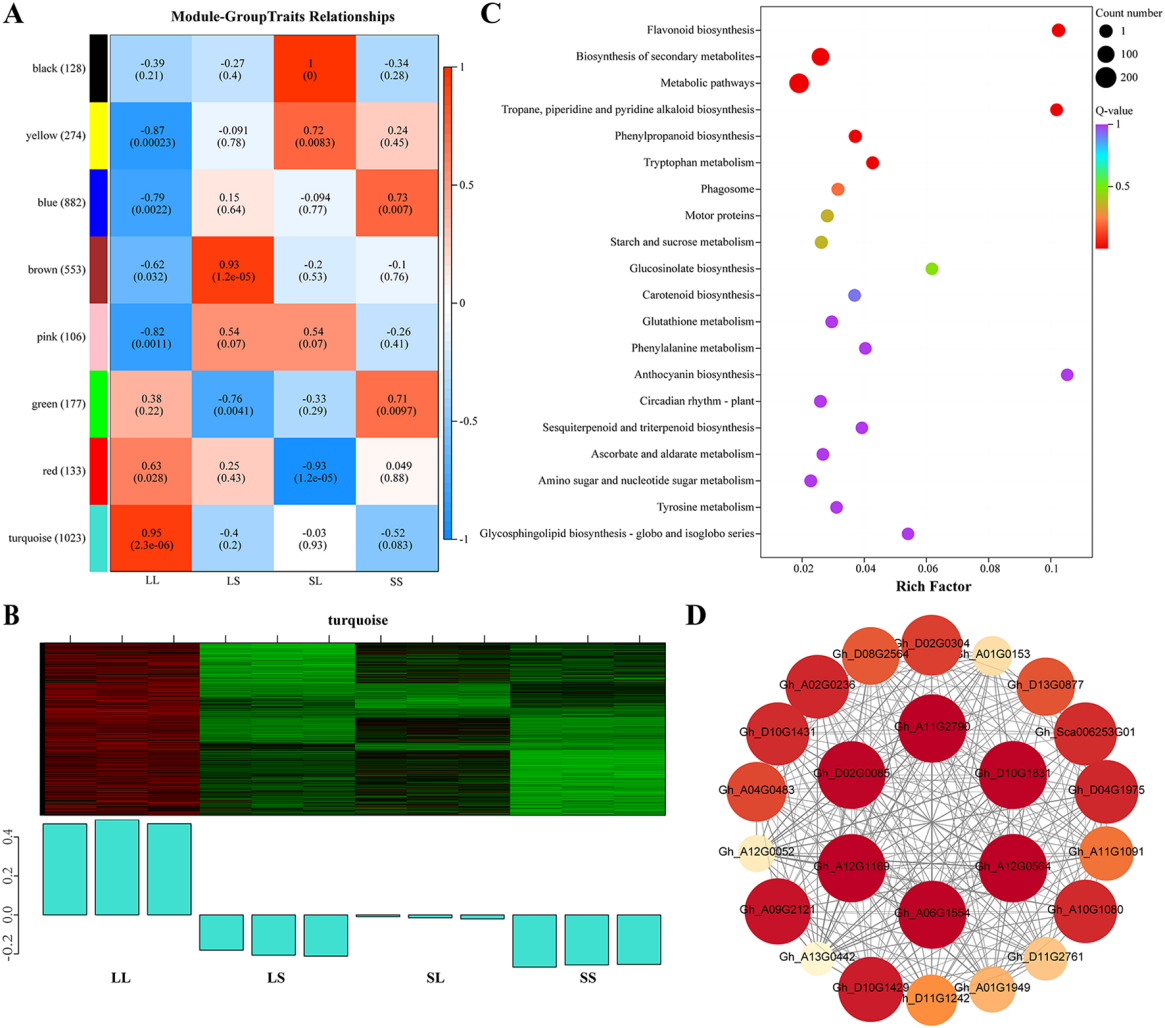
Analysis of weighted gene co-expression networks of DEGs under different photoperiods at the seedling stage. **(A)** Correlation heatmap of gene co-expression network modules and treatments. **(B)** Gene expression characteristics for the turquoise module in different samples. The heatmap above displays the expression patterns of co-expressed genes, while the bar graph below illustrates the expression patterns of these co-expressed genes. **(C)** KEGG analysis of differential genes in the turquoise module. **(D)** A co-expression network is based on the correlations of DEGs in the turquoise module. The size and color depth of the nodes represent the degree of connection of that gene within the module. Detailed information on the differentially expressed genes is provided in **Supplementary Table S4**.

To identify key genes responsive to long-day treatment during the seedling stage in cotton, weighted gene co-expression network analysis (WGCNA) was performed on the DEGs from the three pairwise comparisons. Genes exhibiting a correlation coefficient greater than 0.85 and conforming to a scale-free topology were grouped into modules using a soft threshold power of 5, resulting in eight distinct modules (**Figure 4A**). Among these, the turquoise module contained the largest number of DEGs (1,023), while the pink module contained the fewest (106). Correlation analysis between module eigengenes and treatments revealed that the turquoise module was significantly positively correlated with the LL treatment, whereas the yellow and pink modules were significantly negatively correlated with LL (**Figure 4A**). Furthermore, DEGs within the turquoise module were significantly upregulated under LL treatment compared to LS, SL, and SS treatments (**Figure 4B**), suggesting these genes respond strongly to long-day conditions during the seedling stage and may play a role in promoting flowering in cotton.

KEGG enrichment analysis revealed that the DEGs within the turquoise module are significantly enriched in the “Flavonoid biosynthesis” pathway. Additionally, these genes are associated with pathways including “Tropane, piperidine and pyridine alkaloid biosynthesis,” “Starch and sucrose metabolism,” “Carotenoid biosynthesis,” and “Circadian rhythm – plant” (**Figure 4C**). The co-expression network of DEGs in this module was visualized, and the top six hub genes were identified based on connectivity metrics (**Figure 4D**). These hub genes include those encoding galactan beta-1,4-galactosyltransferase (*GALS1*) (Gh_A11G2790), cellulose synthase-like protein D5 (*CSLD5*) (Gh_A12G1169), protein NRT1/PTR FAMILY 5.1 (*NPF5.1*) (Gh_A06G1554), and formin-like protein 2 (*FLP2*) (Gh_D02G0085). Notably, *FKF1* (Gh_A09G2121), a core gene in the photoperiodic flowering regulation network, also exhibits very high connectivity within this module (**Figure 4D**).

The co-expression network also included several transcription factors and DEGs associated with light response and hormone signaling pathways. Notably, two TIFY transcription factors (Gh_D08G2564 and Gh_A01G0153), which are closely linked to jasmonic acid signaling regulation, were identified. Additionally, a MYB transcription factor (Gh_A01G1949) and two MADS-MIKC transcription factors (Gh_A13G0442 and Gh_D13G0877), known to regulate flowering, were present. Genes encoding photoperiod-related *LWD1* (Gh_D04G1975 and Gh_A04G0483) and gibberellin signaling-related *GID1* (Gh_D11G2761, Gh_A11G1091, Gh_A12G0052, and Gh_D11G1242) were also detected. Furthermore, six genes encoding chalcone synthase (*CHS*) were identified, underscoring the central role of flavonoid biosynthesis within this module (**Figure 4D**).

### 3.4 Metabolic analysis

**Figure 5:**
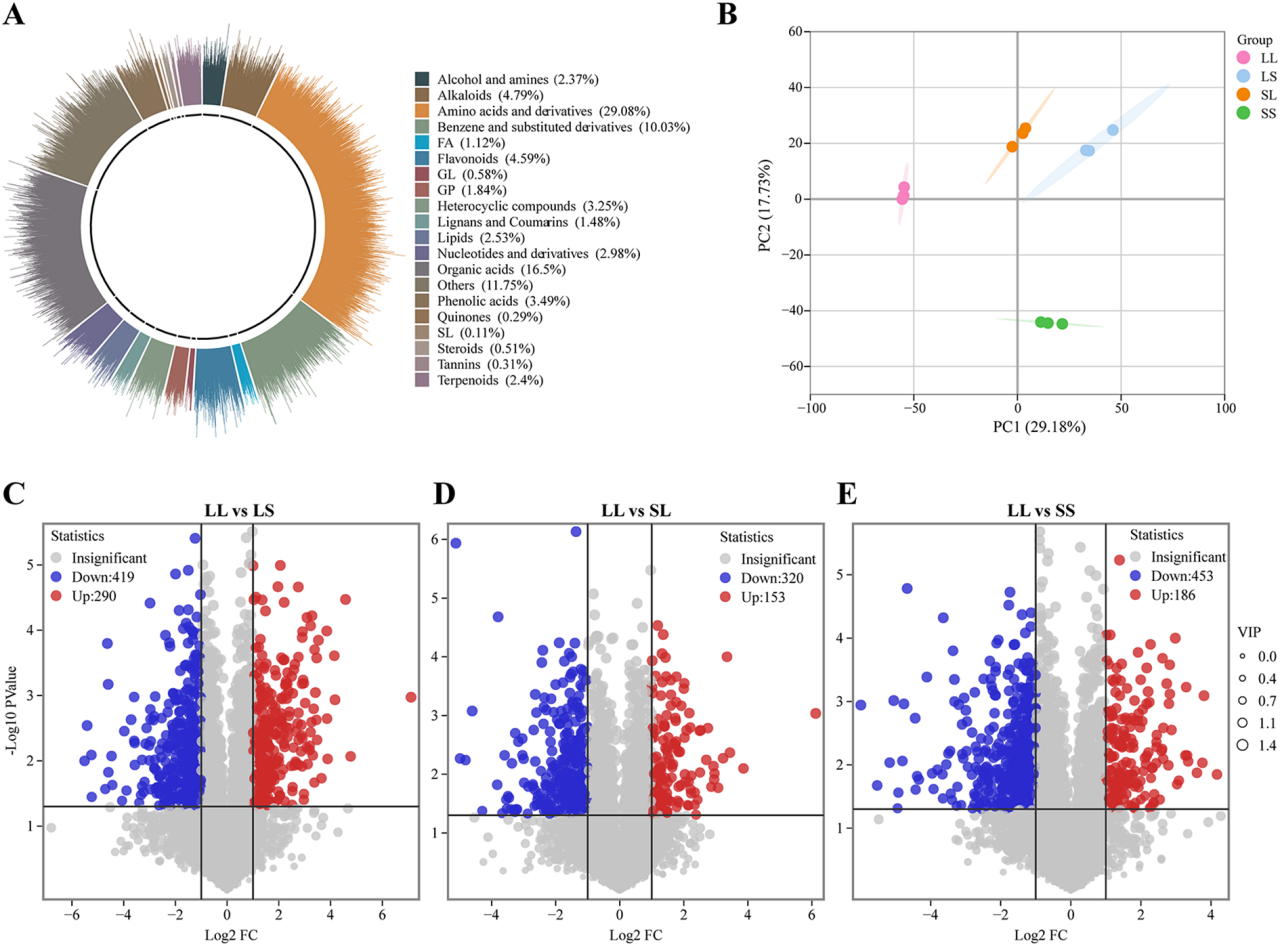
Metabolome analysis of cotton leaves. **(A)** Metabolome chemical structure classification proportion statistics. **(B)** Principal component analysis of metabolome data. **(C–E)** Volcano plot for different treatment groups.

To further investigate the effects of photoperiod treatments during the seedling stage on cotton metabolites, a non-targeted metabolomics analysis was performed on cotton leaves using a UPLC-MS/MS platform. A total of 4,467 metabolites were detected across 12 samples (**Supplementary Table S3**). Based on their structural characteristics, these metabolites were categorized into 20 chemical classes (**Figure 5A**), with the predominant groups being amino acids and derivatives (29.08%), organic acids (16.50%), benzene and substituted derivatives (10.03%), alkaloids (4.79%), and flavonoids (4.59%). Principal component analysis (PCA) demonstrated distinct clustering of samples according to treatment groups, clearly separating the four photoperiod conditions (**Figure 5B**). Differentially expressed metabolites (DEMs) were identified using the thresholds |log_2_Fold Change| ≥ 1, VIP > 1, and p-value < 0.05. Compared to the LS treatment, the LL treatment exhibited 709 DEMs, including 290 upregulated and 419 downregulated metabolites (**Figure 5C**). In the LL vs. SL comparison, 473 DEMs were detected (153 upregulated, 320 downregulated) (**Figure 5D**), while 639 DEMs (186 upregulated, 453 downregulated) were identified in LL vs. SS (**Figure 5E**).

**Figure 6:**
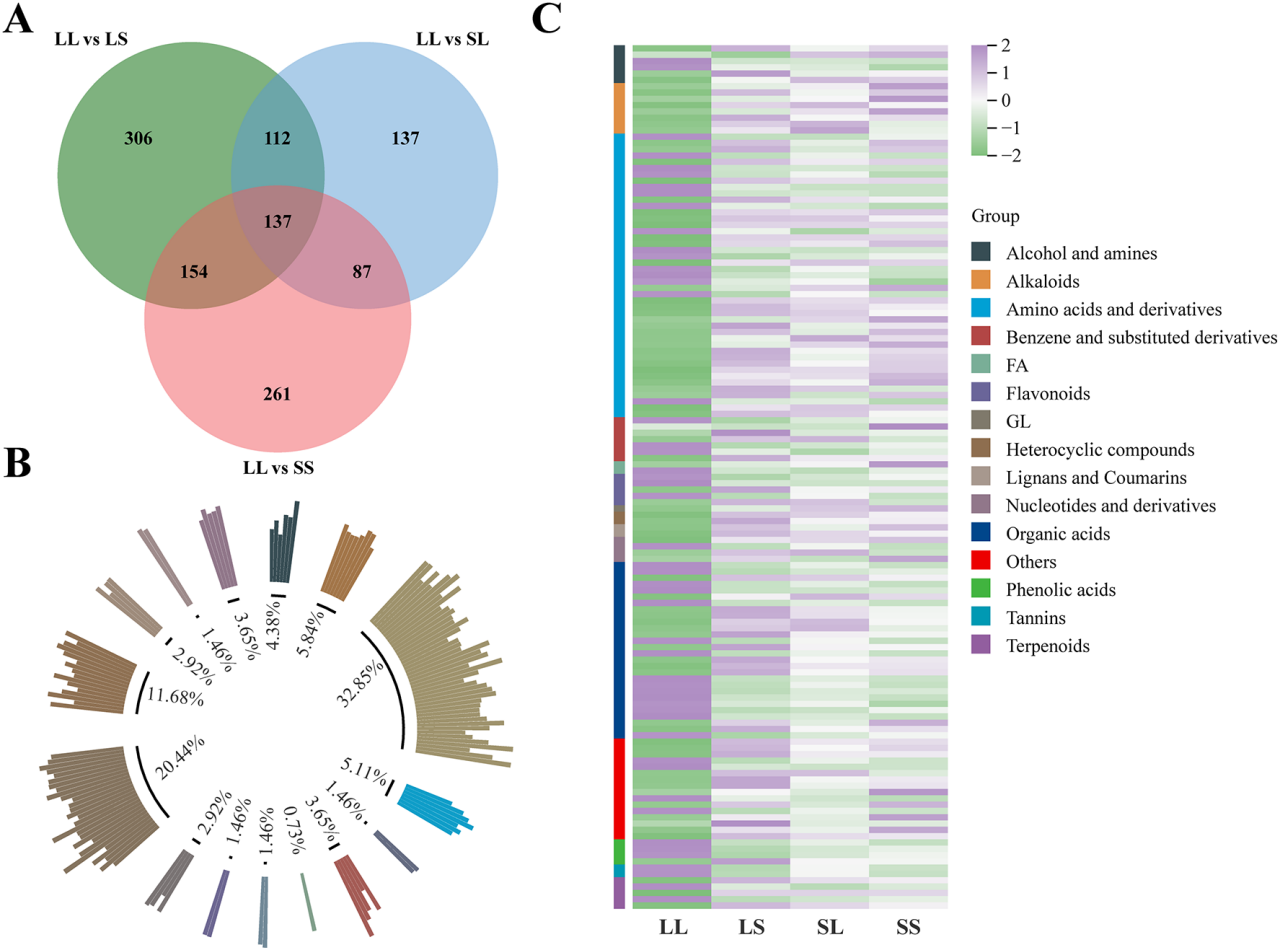
Metabolome difference analysis. **(A)** Venn diagram analysis of DEMs. **(B)** The DEMs common to each treatment group were classified and counted according to their chemical structures. **(C)** DAM’s heat map analysis.

Among all differentially expressed metabolites (DEMs), 137 were commonly identified across the three comparison groups (**Figure 6A**). These shared DEMs were classified into 15 structural categories (**Figure 6B**), with amino acids and derivatives representing the largest group (32.85%), followed by organic acids (20.44%), alkaloids (5.84%), benzene and substituted derivatives (5.11%), alcohols and amines (4.38%), and flavonoids (3.65%). Compared to the LS, SL, and SS treatments, the majority of these common DEMs were downregulated under the LL treatment (**Figure 6C**). This downregulation encompassed alkaloids, glycosides (GL), heterocyclic compounds, lignans and coumarins, most amino acids and derivatives, and other metabolites. Notably, glycosides were most abundant in SL and SS treatments, whereas heterocyclic compounds, lignans, and coumarins were enriched in the LS treatment. Conversely, tannins, most flavonoids, and phenolic acids were significantly upregulated in the LL treatment (**Figure 6C**).

### 3.5 Integrative analysis of transcriptome and metabolome

**Figure 7:**
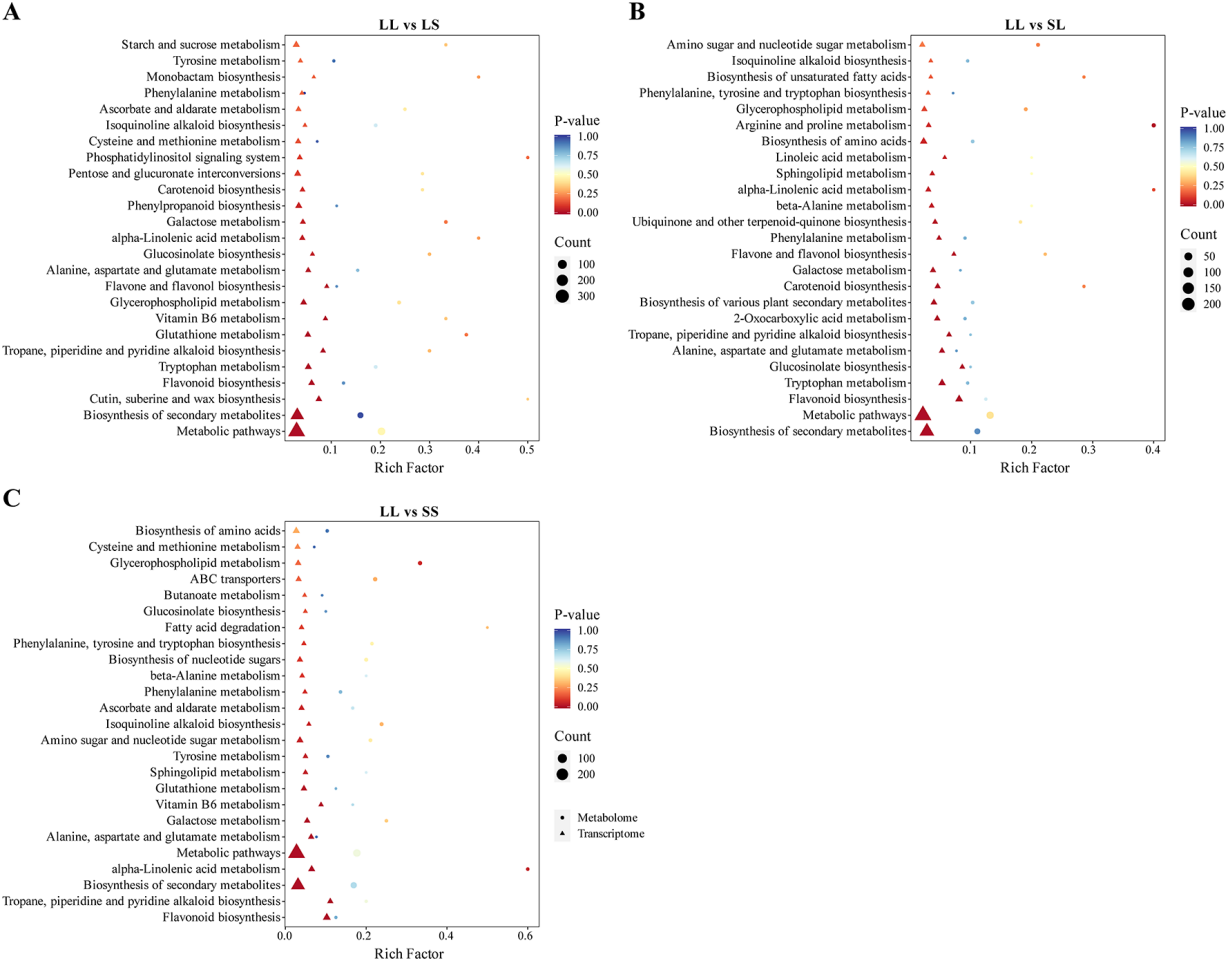
KEGG pathway enrichment of DEGs and DEMs combined. **(A)** KEGG enrichment bubble diagram in LL vs LS. **(B)** KEGG enrichment bubble diagram in LL vs SL. **(C)** KEGG enrichment bubble diagram in LL vs SS.

To comprehensively elucidate the impact of different photoperiod treatments during the seedling stage on major biochemical pathways in cotton, we performed a joint analysis of transcriptomic and metabolomic data to identify key differentially expressed genes (DEGs) and metabolites (DEMs). We focused on overlapping pathways between transcriptional and metabolic levels and conducted KEGG enrichment analysis on pathways annotated by both omics datasets (**Figure 7**). In the LL vs. LS comparison, the main enriched KEGG pathways included “Cutin, suberin and wax biosynthesis,” “Flavonoid biosynthesis,” “Glutathione metabolism,” “Glycerophospholipid metabolism,” and “Tryptophan metabolism” (**Figure 7A**). For LL vs. SL, DEGs and DEMs were primarily enriched in “Flavonoid biosynthesis,” “Glucosinolate biosynthesis,” “Alanine, aspartate and glutamate metabolism,” “2-Oxocarboxylic acid metabolism,” and “Ubiquinone and other terpenoid-quinone biosynthesis” pathways (**Figure 7B**). In the LL vs. SS comparison, significant enrichment was observed in “Flavonoid biosynthesis,” “α-Linolenic acid metabolism,” “Phenylalanine metabolism,” “ABC transporters,” and “Fatty acid degradation” (**Figure 7C**). Notably, the pathways “Glycerophospholipid metabolism,” “α-Linolenic acid metabolism,” and “Flavonoid biosynthesis” were significantly enriched in all three comparisons. Consequently, these three pathways were identified as key shared biochemical pathways responsive to photoperiod treatments during cotton seedling development.

#### 3.5.1 Glycerophospholipid metabolism

**Figure 8:**
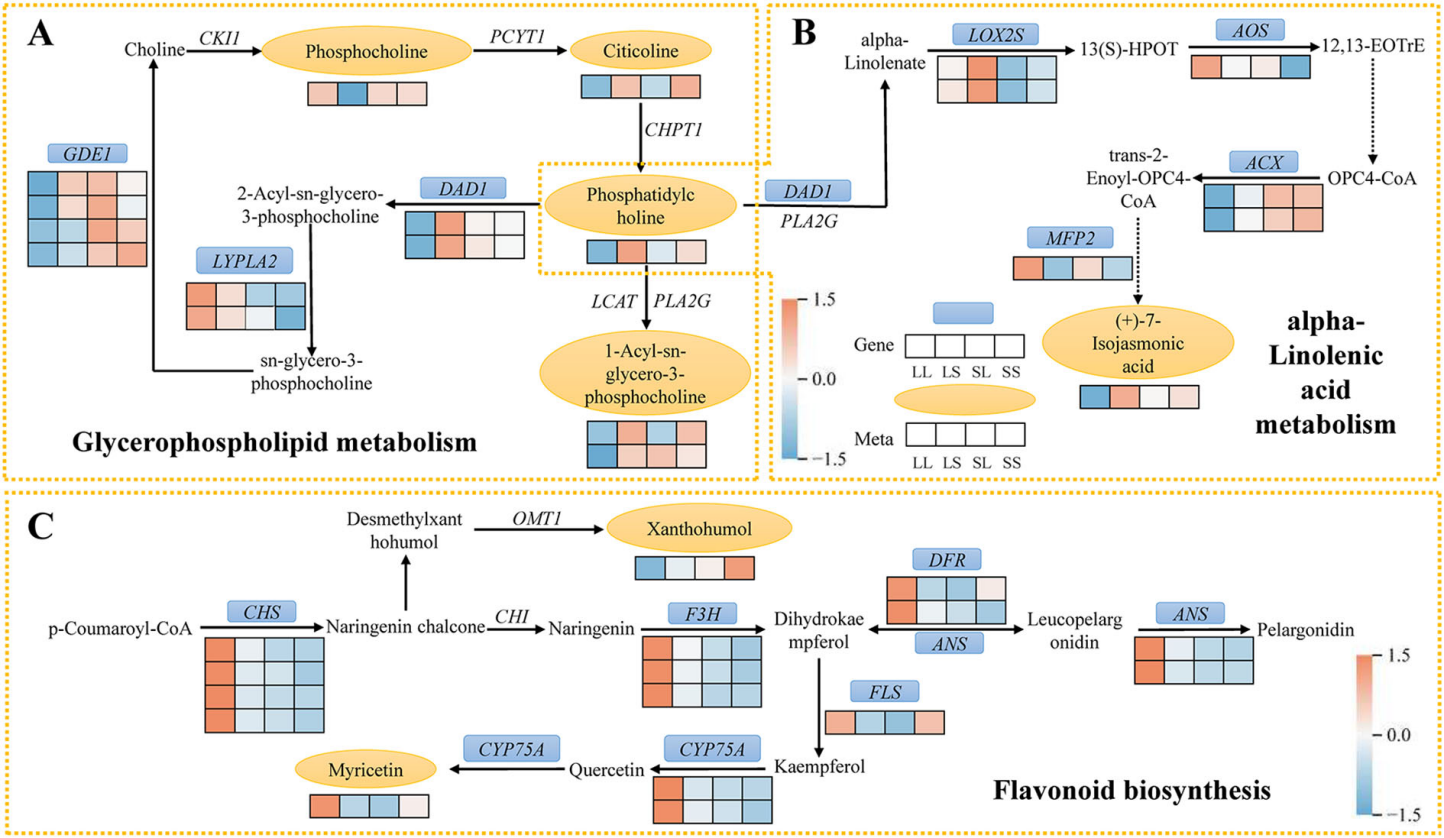
Schematic diagram of the critical pathway. **(A)** Glycerophospholipid metabolism. **(B)** alpha-Linolenic acid metabolism. **(C)** Flavonoid biosynthesis. The blue box and yellow oval highlight the shared DEGs and DEMs. Red and blue indicate the up- and down-regulation of shared DEGs and DEMs.

In the glycerophospholipid metabolism pathway, eight DEGs and five DEMs were identified (**Figure 8A**). Compared to the LS treatment, phosphocholine levels were significantly elevated under the LL treatment. In contrast, the levels of citicoline, phosphatidylcholine, and 1-acyl-sn-glycero-3-phosphocholine were reduced in the LL group. Furthermore, expression of DEGs encoding *DAD1* and *GDE1* was downregulated in LL compared with LS, SL, and SS treatments, whereas DEGs encoding *LYPLA2* showed upregulated expression under LL treatment.

#### 3.5.2 alpha-Linolenic acid metabolism

In the alpha-Linolenic acid metabolism pathway, a total of eight differentially expressed genes (DEGs) and two differentially expressed metabolites (DEMs) were identified (**Figure 8B**). Notably, phosphatidylcholine participates not only in glycerophospholipid metabolism but also serves as a precursor for α-linolenic acid synthesis, which is catalyzed by *DAD1*. The DEGs encoding *LOX2S* were upregulated exclusively in the LL vs. SL comparison, whereas DEGs encoding *AOS* were significantly upregulated only in LL vs. SS. Compared to LS, SL, and SS treatments, DEGs encoding *ACX* and the metabolite (+)-7-iso-jasmonic acid were downregulated under LL treatment. Additionally, DEGs encoding *MFP2* were upregulated in both LL vs. LS and LL vs. SS comparisons.

#### 3.5.3 Flavonoid biosynthesis

A total of 14 differentially expressed genes (DEGs) and 2 differentially expressed metabolites (DEMs) were identified in the flavonoid biosynthesis pathway (**Figure 8C**). Most DEGs were significantly upregulated in the LL treatment compared to the LS, SL, and SS treatments. This included CHS-related DEGs responsible for converting p-Coumaroyl-CoA to Naringenin chalcone, as well as DEGs encoding *DFR* and *ANS*. DEGs associated with *CYP75A*, which catalyzes the conversion of Quercetin to Myricetin, were also expressed at higher levels under LL treatment, leading to a significant increase in Myricetin content. DEGs encoding *F3H* were upregulated only in the LL vs. SL and LL vs. SS comparisons, while DEGs encoding *FLS* were upregulated exclusively in the LL vs. LS and LL vs. SL groups. Additionally, the metabolite Xanthohumol was significantly elevated in the SS treatment but markedly decreased in the LL treatment.

**Figure 9:**
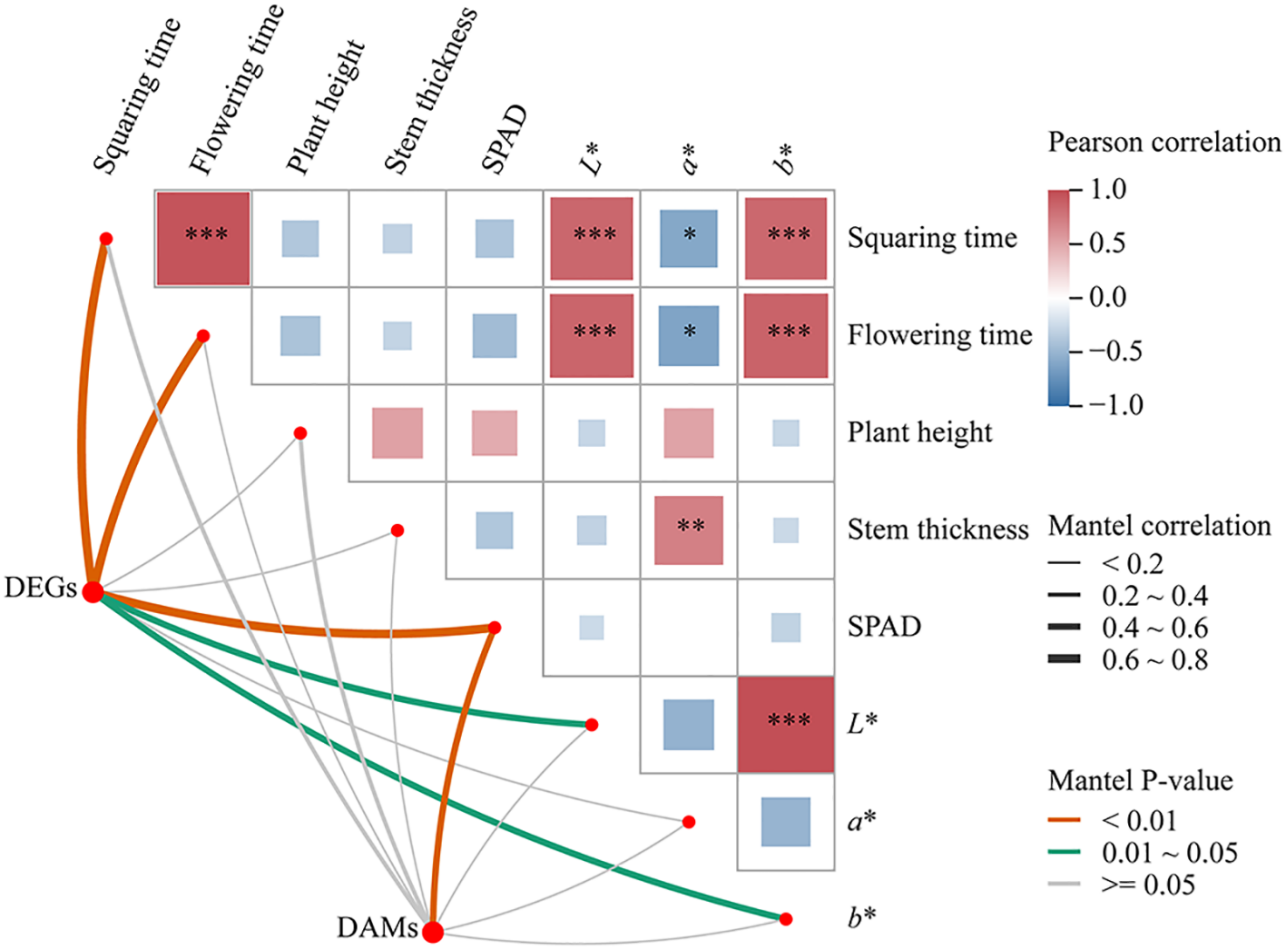
Mantel test of DEGs and DEMs, and plant growth characteristics in key pathways *, p < 0.05; **, p < 0.01; ***, p < 0.001.

A Mantel test (**Figure 9**) was performed to assess the relationships among the relative expression levels of differentially expressed genes (DEGs) and differentially expressed metabolites (DEMs) in the three key metabolic pathways, alongside cotton growth parameters. The correlation analysis revealed that cotton growth traits, including bud development time and flowering time, were significantly positively correlated with leaf color parameters L^*^ and b^*^, and significantly negatively correlated with a^*^. DEGs associated with the key metabolic pathways showed highly significant correlations with bud development time, flowering time, and SPAD values, as well as significant correlations with L^*^ and b^*^. In contrast, DEMs exhibited significant correlation only with the SPAD value.

### 3.6 RT-qPCR validation of cotton transcriptome under different photoperiod treatments at the seedling stage

**Figure 10:**
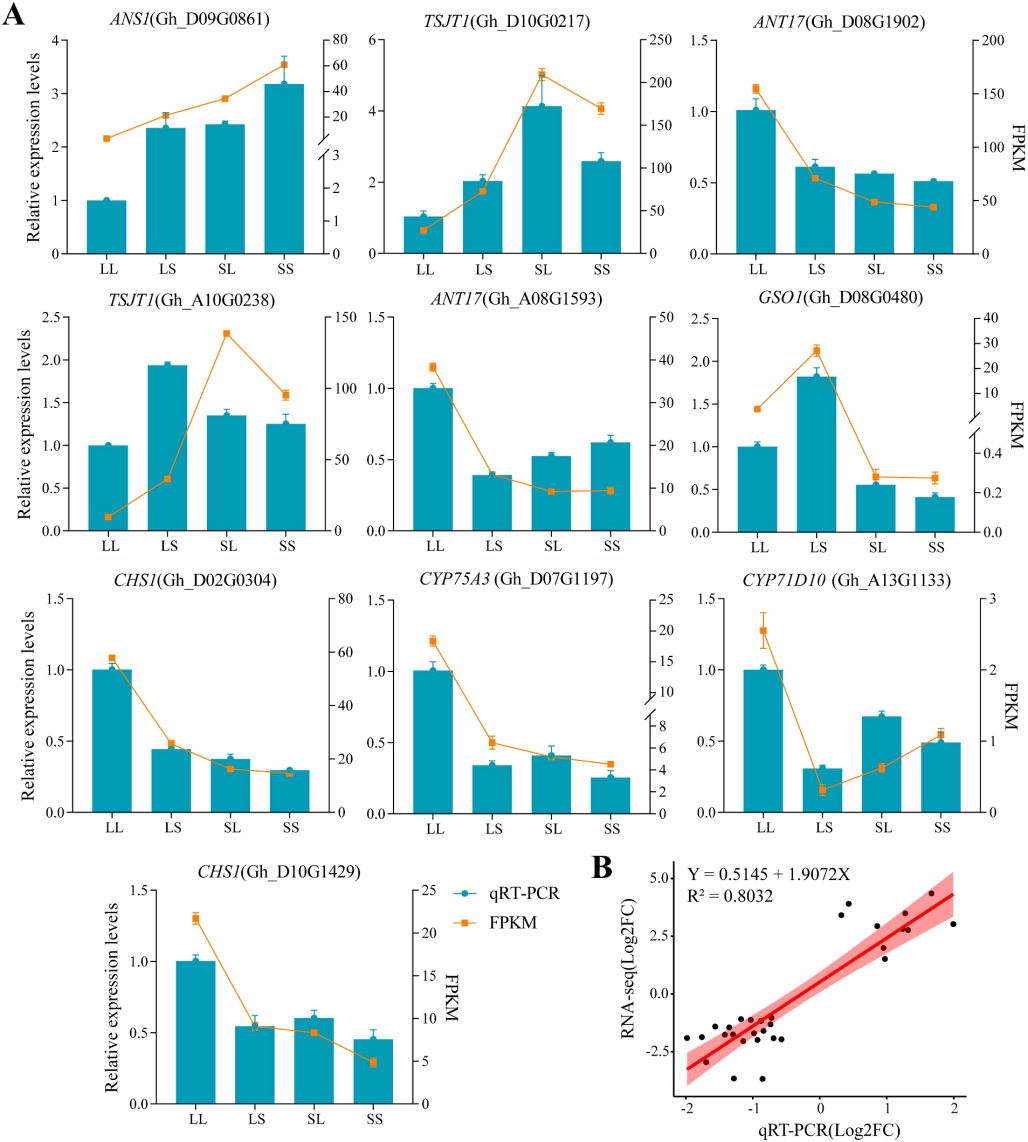
QRT-PCR validation. The data shown is the mean ± standard error.

To verify the accuracy of the transcriptome data, ten differentially expressed genes (DEGs) were randomly selected for quantitative real-time PCR (qRT-PCR) analysis. The primer sequences used for qRT-PCR are listed in **Supplementary Table S1**. The expression patterns detected by qRT-PCR were generally consistent with those obtained from the transcriptome analysis (**Figure 10A**). Regression analysis between qRT-PCR and transcriptome data yielded a coefficient of determination (R²) greater than 0.8 (**Figure 10B**), confirming the reliability of the transcriptome sequencing results.

## 4 Discussion

### 4.1 Effect of photoperiod on cotton growth and development

Photoperiod, a crucial environmental factor regulating plant growth and development, influences multiple stages ranging from photosynthetic efficiency and nutrient accumulation to reproductive transition (Wang et al., 2024a). Although cultivated cotton (*Gossypium hirsutum* L.) is reported to have lost photoperiod sensitivity during domestication (Zhang et al., 2016), our study demonstrates that long-day treatment (LL) during the seedling stage significantly accelerates flowering and blooming, advancing these events by 20 and 17 days, respectively, compared to short-day treatment (SS) (**Figures 1A–C**). This suggests that photoperiod length at specific developmental stages still influences cotton flowering time. Interestingly, the mixed photoperiod treatments (LS and SL) resulted in distinct flowering phenotypes, with the LS group flowering earlier than the SL group. This observation suggests that a ‘phase-sensitive window’ may exist during the seedling stage of cotton, during which the perception of photoperiodic signals is particularly effective in triggering flowering. A previous study has shown that cotton exhibits distinct transcriptional profiles in response to photoperiod between the first and fifth true leaf stages, each playing a crucial role in regulating flowering time (Pan et al., 2024). A similar phenomenon has also been reported in the short-day plant *Vigna angularis* (adzuki bean), where short-day treatment during 5–15 days after germination significantly promotes flowering (Dong et al., 2024), further supporting the key role of an early developmental phase-sensitive window in photoperiodic response in cotton.

Beyond regulating reproductive development, photoperiod treatments significantly affected cotton morphology. Specifically, plants subjected to long-day treatment (LL) exhibited greater height compared to those under short-day treatment (**Figure 1D**). This suggests that extended photoperiods enhance photosynthesis and carbon assimilation, supplying more energy and photosynthates to support cell elongation and stem growth (Wang et al., 2024b). Photoperiod also significantly influenced cotton leaf color. During early development, LL and LS treatments resulted in higher a^*^ values and lower b^*^ values (**Figures 1H, I**), indicating deeper red and blue hues. This change may reflect increased accumulation of secondary metabolites, including flavonoids and anthocyanins, induced by photoperiod. Consequently, leaf color alterations, as direct indicators of physiological status, may serve as valuable phenotypic markers for photoperiod-mediated metabolic regulation. Notably, the transfer of plants to natural outdoor conditions introduces environmental variables—including light spectrum, intensity, and temperature—that may confound the direct effects of the initial photoperiod treatments. Therefore, our conclusions are specifically framed to address the regulatory memory established during the seedling stage.

### 4.2 Effect of photoperiod on the transcriptome and metabolome of cotton

In recent years, advances in high-throughput omics technologies have facilitated widespread use of transcriptomics and metabolomics to unravel complex physiological and molecular regulatory mechanisms in plants. Transcriptomic analysis revealed that differentially expressed genes (DEGs) under varying photoperiod treatments were primarily enriched in pathways related to flavonoid and alkaloid biosynthesis, exopolysaccharide biosynthesis, and alanine, aspartate, and glutamate metabolism (**Figure 3**). These pathways are crucial for plant responses to environmental cues, reproductive organ development, and energy metabolism regulation (Su et al., 2024; Li et al., 2023). Metabolomic analysis further confirmed that photoperiod treatment significantly reprograms cotton metabolism. A total of 137 common differentially expressed metabolites (DEMs) were identified across the LL vs. LS, LL vs. SL, and LL vs. SS comparisons. These included amino acids and derivatives, organic acids, alkaloids, benzene and substituted derivatives, alcohols and amines, and flavonoids (**Figure 6**). Plant-derived metabolites are known to influence flowering time by either promoting or delaying its onset (Chakraborty et al., 2022). Notably, flavonoids significantly modulate flowering time across multiple species by regulating flowering-related genes, influencing hormone signaling pathways, and interacting with photoperiodic cues (Li et al., 2024; Zhou et al., 2024). These findings are fully supported by our current study.

Weighted Gene Co-expression Network Analysis (WGCNA) identified gene modules and key genes associated with photoperiod responses. Notably, the turquoise module showed a strong correlation with the long-day treatment (LL) (**Figure 4A**). Multiple photoperiod- and flowering-related genes were enriched within this module (**Figure 4D**), whose functions have been extensively characterized in model plants. Among these, FLAVIN-BINDING KELCH REPEAT F-BOX 1 (*FKF1*) functions as a blue light receptor and E3 ubiquitin ligase, with its expression modulated by photoperiod signals and circadian rhythms. Under long-day conditions, *FKF1* forms a complex with GIGANTEA (GI) protein, enhancing the stability and transcriptional activity of CONSTANS (CO), which in turn induces FLOWERING LOCUS T (*FT*) expression (Gao et al., 2025; Hwang et al., 2019; Song et al., 2012). Recent studies have confirmed that the cotton homolog *GhFKF1* performs similar functions, showing significant upregulation under long-day conditions and regulating downstream flowering-related gene expression (Pan et al., 2024). In our study, *GhFKF1* expression was significantly elevated in the LL treatment and co-expressed with multiple flowering and hormone signaling genes, including *GID1* and MADS-MIKC transcription factors (**Figure 4D**). Previous studies have shown that in maize, MADS-MIKC genes (*ZMM4* and *ZMM15*) in the shoot apical meristem (SAM) are significantly upregulated following the floral transition (Danilevskaya et al., 2008). In Arabidopsis, the flowering repressor FLOWERING LOCUS C (*FLC*) expressed in the SAM directly represses the transcriptional activity of the MADS-MIKC gene *SOC1*, thereby delaying flowering (Searle et al., 2006). However, in this study, transcriptomic and metabolomic analyses were conducted solely on leaf tissues. Although leaves are the site of florigen production in the photoperiodic flowering pathway, the actual initiation of flowering occurs in the SAM (Yang et al., 2024). Thus, transcriptional changes observed in leaves may not fully reflect the regulatory processes occurring in the SAM. Therefore, future studies should focus on examining the expression of key regulatory factors in the SAM to gain a comprehensive understanding of the flowering induction process. Moreover, *FKF1* in Arabidopsis can directly interact with DELLA proteins, promoting their ubiquitination and degradation, which enhances plant sensitivity to gibberellin (GA) signaling and promotes flowering (Yan et al., 2020). *GID1*, as the GA receptor, similarly facilitates DELLA protein degradation. Based on this, we hypothesize that in cotton, *GhFKF1* may synergistically regulate the *GID1*-DELLA module to integrate circadian clock and hormonal signals, forming a multi-pathway regulatory mechanism controlling flowering timing.

### 4.3 Effects of photoperiod on glycerophospholipid metabolism, alpha-Linolenic acid metabolism, and flavonoid biosynthesis in cotton

Combined transcriptomic and metabolomic analyses in this study revealed the concurrent enrichment of three key pathways: glycerophospholipid metabolism, alpha-linolenic acid metabolism, and flavonoid biosynthesis. Glycerophospholipid metabolism primarily governs the synthesis and degradation of membrane lipids. Its core component, phosphatidylcholine (PC), serves as both a major membrane lipid and a precursor of fatty acids essential for jasmonic acid (JA) biosynthesis (Kumari et al., 2015). Under LL treatment, levels of PC and its precursor cytidine diphosphate (citicoline) were significantly decreased, alongside marked downregulation of *DAD1*, a gene involved in phospholipid hydrolysis (**Figure 8A**). This likely reduced the availability of substrates for JA biosynthesis. Alpha-linolenic acid metabolism constitutes the central pathway for JA biosynthesis (Holtsclaw et al., 2024). Previous studies have demonstrated that JA binds to its receptor *COI1*, promoting degradation of JAZ proteins, which release APETALA2-type transcription factors that suppress *FT* expression, consequently delaying flowering (Zhai et al., 2015). Moreover, JA and gibberellin (GA) signaling pathways interact antagonistically; elevated JA levels suppress GA biosynthesis (Heinrich et al., 2013), causing DELLA protein accumulation and consequent flowering inhibition. In our study, LL treatment downregulated JA biosynthesis-related *ACX* genes and upregulated the JA degradation-related *MFP2* gene, corresponding with decreased levels of the JA precursor (+)-7-isojasmonic acid (**Figure 8B**). These findings suggest that photoperiod modulates JA biosynthesis via lipid metabolism regulation, thereby influencing flowering time. Flavonoids, a class of widespread plant secondary metabolites, contribute not only to pigment formation but also to key processes in growth and development, including flowering time regulation (Li et al., 2024). Studies have shown that long-day conditions induce flavonoid biosynthesis genes, promoting floral bud initiation and growth in *Liriodendron chinense* (Hussain et al., 2022). Here, several key flavonoid biosynthesis genes—including *CHS*, *F3H*, *DFR*, *ANS*, and *CYP75A*—were upregulated under LL treatment, accompanied by increased accumulation of the metabolite myricetin (**Figure 8C**). This supports the conserved and active role of this pathway in cotton’s photoperiodic response. In the WGCNA analysis, the expression of Tify and MYB-related transcription factors was significantly upregulated in the LL treatment group (**Figure 4D**). Among them, Tify family proteins, especially JAZ proteins, act as key negative regulators in the jasmonic acid (JA) signaling pathway by interacting with MYC transcription factors such as *MYC2*, thereby repressing the expression of JA-responsive genes (Lu et al., 2025). Additionally, MYB transcription factors can directly bind to the promoters of flavonoid-related genes, including *FLS*, *F3H*, and *CHS*, activating their transcription and positively regulating flavonoid accumulation (Zhao et al., 2022).

In summary, photoperiod treatment likely regulates cotton’s transition from vegetative to reproductive growth via a dual mechanism: promoting flavonoid biosynthesis while suppressing jasmonic acid (JA) accumulation, coordinated with circadian rhythm signaling. It is important to acknowledge that this study presents a preliminary regulatory framework derived from integrated transcriptomic and metabolomic analyses, which nonetheless has inherent limitations. While non-targeted metabolomics facilitates comprehensive metabolic profiling, uncertainties persist regarding the structural annotation and quantitative accuracy of certain metabolites (Hendrik et al., 2016). Furthermore, our study employed only a single sampling time point and focused on an early-maturing cotton cultivar that is more sensitive to photoperiod. It remains unclear whether the dynamic changes in gene expression and metabolite accumulation, as well as the responses to day length, are consistent across other cultivars. Therefore, future research should incorporate multiple sampling time points, trials involving diverse cultivars, hormone level measurements, and functional validation of key genes to comprehensively elucidate the roles of these metabolic regulatory modules in the development of early maturity traits in cotton.

## 5 Conclusions

This study systematically elucidates the molecular mechanisms underlying photoperiodic regulation of flowering time in cotton by integrating phenotypic, transcriptomic, and metabolomic analyses under varied photoperiod treatments during the seedling stage. Results demonstrate that long-day treatment significantly accelerates both the initiation and timing of flowering in cotton. Transcriptomic analyses reveal that long-day treatment markedly upregulates the core photoperiod gene *GhFKF1*, which co-expresses with flowering-related genes, including MADS-MIKC transcription factors and the gibberellin receptor *GID1*. This suggests a coordinated regulation of flowering via interactions between circadian rhythm and hormonal signaling pathways. Metabolomic profiling shows that photoperiod treatments reprogram cotton’s metabolome, notably leading to significant accumulation of flavonoid metabolites under long-day conditions. Integrated multi-omics analysis identifies three metabolic pathways—glycerophospholipid metabolism, alpha-linolenic acid metabolism, and flavonoid biosynthesis—as significantly affected by photoperiod treatment. Under long-day light conditions, the expression of genes involved in jasmonic acid biosynthesis and the accumulation of its precursor compounds were reduced, while the expression of flavonoid biosynthesis-related genes was upregulated. These findings suggest that photoperiod may regulate flowering by simultaneously suppressing jasmonic acid signaling and activating the flavonoid pathway. Overall, long-day photoperiod during the seedling stage appears to coordinate hormone signaling and secondary metabolism through the upregulation of *GhFKF1* expression, thereby promoting the initiation of flowering in cotton. This study provides multi-omics evidence elucidating the regulatory role of photoperiod in the reproductive transition of cotton, demonstrating a strong correlation among gene expression, metabolite accumulation, and flowering time, and offering novel insights for future functional validation studies.

## Data Availability

The raw sequence data reported in this paper have been deposited in the Genome Sequence Archive (Genomics, Proteomics & Bioinformatics 2021, Chen et al., 2021) in National Genomics Data Center (Nucleic Acids Res 2022, CNCB-NGDC Members and Partners, 2022), China National Center for Bioinformation / Beijing Institute of Genomics, Chinese Academy of Sciences (GSA: CRA029661) that are publicly accessible at https://ngdc.cncb.ac.cn/gsa.

## Supplementary material

Supplementary Table 3 Metabolites from four groups of samples.

Supplementary Table 4 All_DEG.summary.
